# Medicines quality assurance to fight antimicrobial resistance

**DOI:** 10.2471/BLT.17.199562

**Published:** 2017-12-01

**Authors:** Jude Nwokike, Aubrey Clark, Phillip P Nguyen

**Affiliations:** a The Promoting the Quality of Medicines Program, US Pharmacopeial Convention, 12601 Twinbrook Parkway, Rockville, Maryland, 20852, USA.; b United States Pharmacopeial Convention, International Public Policy and Regulatory Affairs, Rockville, USA.

Antimicrobial resistance is increasingly the focus of global attention. The adoption of resolution 68.7 at the 68th World Health Assembly^1^ was pivotal in the ongoing fight against antimicrobial resistance, as the resolution urges Member States to develop national action plans to fight antimicrobial resistance by 2017. To date, 67 Member States, out of 194, have already developed such plans, while 62 others are in the process of doing so.^2^ These national plans describe national efforts to combat resistance through strategies and interventions that often include strengthening surveillance of priority pathogens, increasing research efforts and optimizing the use of antimicrobials, among others.

This paper suggests that product quality surveillance and other quality assurance measures should be thoroughly addressed in efforts to contain antimicrobial resistance. Including such measures in national action plans and key normative guidance documents for antimicrobial resistance is critical to containment, especially for low- and middle-income countries, where weak regulatory controls may increase the potential for poor-quality antimicrobials to be widely available.

## The role of product quality

In 2017, the World Health Organization (WHO) adopted new and relevant definitions^3^ for classifying poor-quality medicines, clarifying the understanding of substandard medical products as those that fail to meet quality standards and/or specifications, also known as out-of-specification products. Testing against public standards helps ensure the identity, strength, quality and purity of medicines and is the foundation for regulating their quality. 

Evidence shows that exposure to subtherapeutic levels of an antibiotic can promote development of resistant bacterial strains and increased virulence, threats that can give rise to deadlier infections.^4^ In clinical practice, medication nonadherence is often cited as a driver of antimicrobial resistance.^5^ However, poor-quality medicines impede the efforts of patients who take medicines correctly, creating the same subtherapeutic result and potentially ending in treatment failure.

Use of poor-quality medicines also directly increases mortality and complicates the monitoring and detection of resistance as an epidemiologic driver of poor disease outcomes. One study estimated that in 2013 more than 122 000 malaria deaths in children younger than five years, that is, 3.8% of all deaths in that age group across the 39 countries studied, were associated with consumption of poor-quality antimalarials.^6^

Treatment failure due to poor-quality medicines may be incorrectly attributed to a resistant infection, even in cases where standard treatment guidelines are followed. This is of particular concern in low- and middle-income countries where availability and use of susceptibility testing can be uneven, time-consuming and expensive. A study of malaria outbreaks in eastern Afghanistan and Pakistan in 2002 and 2003 determined that several cases of presumed drug resistance were in fact drug-sensitive infections treated with poor-quality medicines.^7^ Such situation can cause unnecessary switches to more aggressive treatment regimens when a quality-assured first-line treatment could have been successful. Additionally, patients who are switched to new regimens may still encounter problems with the quality of medication.^8^

Although disease-specific data indicate that poor-quality medicines may be a driver in the development of antimicrobial resistance, comprehensive data on the extent to which poor-quality medicines contribute to resistance are lacking. Attempting to determine the relative contribution of other key drivers, such as antimicrobial use in animal production presents similar challenges. However, existing evidence on the use of antimicrobials in animal production has prompted many stakeholders to take proactive measures based on the precautionary principle in public health.^9^ The same rationale should prompt actions to address poor-quality medicines in the fight against such resistance, especially since there are proven methods that can help ensure the quality of medicines.

For the reasons outlined above, any effort to optimize the use of antimicrobials should be accompanied by adequate consideration of the antimicrobial’s quality. How well an antimicrobial works is integrally linked to how well quality is assured, from manufacture to distribution and storage in the supply chain, until the product is used by the patient. Additionally, expanding access to non-quality assured antimicrobials may accelerate the rise of resistance by exposing larger populations to subtherapeutic doses of medicines.

Comprehensive data on the proportion of poor-quality medicines is lacking, but data from WHO estimates that 1 in 10 medical products in low- and middle-income countries is substandard or falsified. In addition, anti-malarials and antibiotics are amongst the most commonly reported substandard and falsified medical products.^10^

Currently, many national action plans on antimicrobial resistance emphasize the importance of improving access to antimicrobials while optimizing their use, but fewer plans include comprehensive measures to ensure the quality of antimicrobials.

## Ensuring antimicrobial quality

Several approaches to ensure antimicrobial quality are being suggested, including those proposed by WHO, the Asia-Pacific Economic Cooperation and the Promoting the Quality of Medicines programme^11^, a programme funded by the United States Agency for International Development (USAID) and implemented by the United States Pharmacopeia. These approaches can be integrated into activities already included in global strategies and national action plans. Table 1 provides examples from Bangladesh, Indonesia and South Africa, of how efforts to address product quality can be incorporated in national action plans on antimicrobial resistance. These examples have been aligned with WHO’s prevent, detect, respond model for addressing poor-quality medicines^3^ and represent only a fraction of the actions that countries could take to address poor-quality medicines (Box 1). Additional work is required to delineate the full range of actions that could be relevant for addressing poor-quality medicines as part of the fight against drug resistance.

**Table 1:** Examples of goals, objectives and activities related to product quality in antimicrobial resistance national action plans from Bangladesh, Indonesia, and South Africa

Country	Policy goal/objective for drug quality in antimicrobial resistance national action plans	Sample activities
Bangladesh	Strengthen regulatory mechanism to ensure access of quality antibiotics	• Ensure good manufacturing practice of antimicrobials in human and animal sectors • Monitor manufacture of quality-assured antimicrobials • Conduct regular post-marketing surveys on the quality of antimicrobials
Indonesia	Regulate post-marketing quality of drugs under the coordination of drug regulatory authority to ensure access to safe and quality antibiotics	• Coordinate quality control by the drug regulatory authority along the pharmaceutical supply chain of antimicrobial agents and active pharmaceutical ingredients • Conduct periodic surveys to estimate the extent of non-prescription sales of antibiotics and active pharmaceutical ingredients and their drivers • Gradually implement one drug regulatory system in human health, animal health and aquaculture
South Africa	Ensure the availability and accessibility of essential drugs to all citizens, to ensure the safety, efficacy and quality of drugs	• Establish national surveillance system for antimicrobial quality • Strengthen laboratory systems to monitor quality assays and pharmacovigilance reporting systems

Source: *Road map of the national action plan of ARC*, Bangladesh;^12^ *National action plan on antimicrobial resistance 2017–2019*, Indonesia;^13^ and *Antimicrobial resistance national strategy framework 2014–2024*, South Africa.^14^

Box 1 **Potential areas of intervention for ensuring the quality of antimicrobials in national action plans on antimicrobial resistance**

**Strengthen the knowledge and evidence base through surveillance and research**

Detection:

- Conduct studies to assess the relationship between availability of poor-quality medicines and presence of drug-resistant microbes

**Optimize the use of antimicrobial medicines in human and animal health**

Prevention:

- Strengthen and optimize registration and market authorization systems within the national medicines regulatory authority
- Promote and operationalize principles of reliance on stringent regulatory authorities and regional harmonization
- Ensure sufficient WHO prequalified sources of medicines
- Ensure compliance with current good manufacturing practices
- Ensure good supply chain practices including proper dispensing
- Procure medicines from WHO prequalified sources

Detection:

- Monitor antimicrobial quality through strategic and risk-based post-marketing surveillance programmes
- Strengthen national quality control laboratories to comply with ISO 170125 or WHO prequalification standards
- Ensure routine inspection of products imported and utilize appropriate screening technologies

Response:

- Use data on medicines quality to inform and take regulatory action
- Develop mechanism(s) for communicating notices of poor-quality antimicrobials with relevant stakeholders

ISO: International Organization for Standardization; WHO: World Health Organization.Source: The World Health Organization’s global action plan on antimicrobial resistance, 2015.^1^

While gaps in antimicrobial resistance surveillance are well-recognized, efforts to strengthen surveillance are often focused on building capacity of microbiology or clinical laboratories and omit efforts to address quality surveillance. However, strengthening quality assurance and regulatory systems, particularly implementing risk-based post-marketing quality surveillance and supporting national quality control laboratories to accurately and reliably verify the quality of antimicrobials, can reinforce surveillance efforts. Ensuring that a quality surveillance system that provides timely and reliable data on drug quality is part of the comprehensive infrastructure for antimicrobial resistance surveillance should be a public health imperative. These systems should work together, especially since product quality issues can signal the risk of potential development of antimicrobial resistance and vice versa.

The Promoting the Quality of Medicines programme, for example, has been working since 2009 to strengthen medicines quality surveillance systems in low- and middle-income countries. This programme has supported regulatory authorities in identifying the presence of poor-quality medicines and taking more than 330 evidence-based enforcement actions, including the recall of products. Data from these quality surveillance activities feed the open access Medicines Quality database, which houses nearly 15 000 medicine-quality test results. These medicines are mostly antimicrobial products from 200 sentinel sites across Africa, Asia and Latin America. This database along with other initiatives, including WHO’s Global Surveillance and Rapid Alert systems, are the bulk of the openly available data on product quality. These complementary efforts need continued investments and ongoing harmonization to collect data that will be important in continuing to assess and monitor antimicrobial quality.

Ensuring that procured medicines meet international quality standards, such as WHO prequalification, is another critical measure that can prevent poor-quality antimicrobials from reaching patients. In addition to procuring from WHO-prequalified sources, working with manufacturers to improve compliance with good manufacturing practices encourages the continuous and reliable availability of quality-assured antimicrobials. This approach has enabled critical antibiotics to be produced, including those necessary for the treatment of tuberculosis and maternal and child health conditions. In particular, the Promoting the Quality of Medicines’ support to more than 50 manufacturers has resulted in WHO prequalification for critical antituberculosis medicines, including rifampicin, kanamycin and moxifloxacin.

Failing to include key quality assurance measures, such as antimicrobial drug quality surveillance, in plans and strategies aimed at combatting antimicrobial resistance risks overlooking a possible contributing factor to resistance. Building quality assurance into the architecture of the response to antimicrobial resistance, including efforts to strengthen surveillance, optimize rational use and improve supply chain and availability, can strengthen and amplify the effect of other containment efforts. Quality-assured medicines must be an essential part of the strategies and plans to address the increase of antimicrobial resistance.
